# In the Twisted Fairytale of Human Agency, Irrational Beliefs Are the Unlikely Heroes

**DOI:** 10.1007/s12115-025-01108-6

**Published:** 2025-05-05

**Authors:** Lisa Bortolotti

**Affiliations:** 1 University of Birmingham, Birmingham, UK; 2 University of Ferrara, Ferrara, Italy

**Keywords:** Agency, Irrationality, Rationality, Illusion, Delusion, Mental health

## Abstract

Agency is our capacity to intervene in the world to pursue our goals. It is commonly assumed that we need to be rational in order to be effective agents, and that rational agency is also something that contributes to our mental health. Indeed, several medical interventions aimed at improving functioning are interpreted as a way to restore rational capacities that had been previously compromised. However, research in cognitive and social psychology has undermined the idea that we are rational agents and has drawn attention to forms of behaviour that are at the same time irrational and instrumental to our wellbeing. Embracing this complexity is vital to gaining a better understanding of why, at least in some contexts, reinstating rationality does not enhance mental health. Moving towards a view of our agency that is less idealised also enables us to develop effective strategies for interacting in a productive way with people who have different beliefs and values from ours, where a productive exchange is one where all parties gain something from the exchange. In this paper, I briefly discuss some cases of irrational belief that support, rather than compromise, agency.

## Rationality and Agency

We expect the things we value to cluster together. In many fairytales, the princess is at the same time beautiful, kind-hearted, and brave. She faces threats to her happiness, but avoids causing unnecessary harm, and is always fearless and graceful. Undesirable features also cluster together in traditional fairytales: the witch is old and ugly, mean, and cowardly too. She gets an unfair advantage over common folks with her magic powers, she deceives and gobbles up innocent children, and she is unpleasant to look at, with her long nose and wrinkled face. Although retellings of traditional fairytales revisit these clusters in creative ways, the assumption that moral virtues and good looks go together is rarely challenged (Widdows 2018).

In traditional approaches to the philosophy and psychology of human cognition, we also cluster all good things together and expect rationality, mental health, and agency to feed on and support each other. When rationality falters, agency is undermined. When rationality is abandoned altogether, mental health is compromised. Let me offer an example of an influential view that relies on these connections. Think about Donald Davidson’s approach to radical interpretation (Davidson 1974), which builds on previous work in the philosophy of the social sciences, such as Dilthey’s and Herder’s examination of what it takes to understand a person who is different from us (Dilthey 1989 [1883]; von Herder 2002 [1774]). The idea behind radical interpretation is that our attempts at understanding people who do not share a language or a culture with us get off the ground only because we assume that all people are by and large rational.

How does this work? Imagine that you are an intrepid anthropologist reaching a secluded tribe on an unexplored island. You don’t have background information about the tribe’s form of life. But when a member of the tribe points to a hopping rabbit and utters: “Gavagai!”, you feel fairly confident in interpreting them as saying: “Look, a rabbit!” (or something similar) because you assume that people interact with their environment as you do with yours. When the environment is shared (e.g., you and the other person both witness the appearance of the same rabbit in the same clearance), it becomes a bridge that makes partial understanding possible. Starting from the person’s reaction to the rabbit hopping away, you can begin to make sense of the other person’s utterances. In radical interpretation, the ascription of beliefs, desires, and other intentional states can be made only against a background of shared rationality.

Davidson invokes the principle of charity in radical interpretation, which has been formulated in numerous ways and had an enormous influence in philosophy, psychology, and the social sciences. We are invited to ascribe to others the beliefs we suppose we would have ourselves if we were in the same circumstances as they are. When it seems to us that a speaker is saying something wrong, something different from what we would say in the same circumstances, then we should doubt the accuracy of our interpretation before ascribing to the speaker an obviously false belief.

In Daniel Dennett’s formulation of the principle, which is the tenet of his famous *intentional stance* (Dennett 1987), the reference to rationality as a constraint on the interpretation and prediction of other agents’ behaviour becomes even more explicit:

[F]irst you decide to treat the object whose behavior is to be predicted as a rational agent; then you figure out what beliefs that agent ought to have, given its place in the world and its purpose. Then you figure out what desires it ought to have, on the same considerations, and finally you predict that this rational agent will act to further its goals in the light of its beliefs. A little practical reasoning from the chosen set of beliefs and desires will in most instances yield a decision about what the agent ought to do; that is what you predict the agent will do. (Dennett 1987, p. 17)

For Davidson and Dennett, it is the assumption that a speaker is rational that guides the interpreter’s attempts at understanding and predicting the speaker’s behaviour. When explanation and prediction of the speaker’s behaviour become difficult, the interpreter can no longer read the speaker’s mind off their behaviour. Sustained difficulties in explaining and predicting behaviour cause puzzlement at first, and disengagement in the long run. Think about how people react when facing views that are very different from their own: “Surely you don’t mean that!”, “Are you delusional?”, or “You must be out of your mind”. The absence of a common ground, when prolonged and systematic, is no longer considered as a translation problem and raises concerns about the speaker’s capacity to exercise rational agency (Bortolotti 2024).

## Agency and Mental Health

Rationality, agency, and mental health go together. They are the beauty, kindness, and courage of the princess in a fairytale of our own making. Epistemic rationality encompasses our capacity to adopt beliefs that are well grounded in evidence and ensures that our existing beliefs remain responsive to external challenges. Practical rationality includes our capacity to act and make decisions for good reasons. Agency is the capacity to intervene in our physical and social world to pursue and achieve our goals, giving shape and direction to our lives. Mental health is a state of emotional, psychological, and social wellbeing, which is preserved in the face of significant difficulties.

As we saw, in the psychological and philosophical literature alike, rationality is often an explicit condition for agency. Repeated failures to interpret and predict a person’s behaviour can be taken to indicate that the person’s agency is compromised, and the mental health of that person is often called into question as a result. Think about the poignant story by Luigi Pirandello, *One, No One, and One Hundred Thousand*, where the protagonist, Vitangelo Moscarda, realises that the way other people see him does not correspond to how he sees himself. There is not just one but many Vitangelo Moscarda! Shaken by this realisation, Vitangelo starts behaving in ways that defy other people’s expectations of him, creating confusion and embarrassment, until most agree that he must have gone mad.

This inference from unpredictability of behaviour to madness due to failures of interpretation and prediction is often discussed in the antipsychiatry movement where psychiatric diagnosis is seen as a means for social control. For Thomas Szasz for instance, the phrase “mental illness” is a misleading metaphor which is applied to the behaviour of people who defy societal expectations, especially those expectations that are highly valued and thus considered as standards of rationality:

[M]ental illness is a myth, whose function it is to disguise and thus render more palatable the bitter pill of moral conflicts in human relations. (Szasz 1960, p. 118)

Even if we do not share this radical picture of the nature of mental illness, it is an interesting fact that the symptoms of mental disorders listed in manuals for psychiatric classification and diagnosis are characterised primarily as failures of rational agency or moral conduct. These perceptions are reflected in commonsense understandings of the people living with a mental health issue as “faulty”. The person living with depression is often viewed as someone who has an excessively negative view of themselves which paralyses them to the point that they stop pursuing the goals they value. The person living with schizophrenia is represented in common discourse and popular culture as someone whose hallucinations and incoherent thoughts fail to represent reality accurately and lead to verbal and nonverbal behaviour that others cannot understand, and that is often feared and contained.

If mental health issues are failures of rationality, then they need to be addressed by restoring rationality. Research on depression has focused on the effects of negative automatic thoughts of the kind “I am no good”, “I am unlovable”, “I cannot do anything right”, which are not justified based on evidence of past behaviour. Although we may all have such thoughts occasionally, in people with depression they tend to be the default explanation for challenging situations, contributing to low self-esteem and helplessness. In the context of talking therapies, such thoughts are often explicitly addressed. In cognitive behavioural therapy, for instance, clients are encouraged to become aware of their negative automatic thoughts, challenge them, and change their behaviour accordingly. In this picture, the irrationality of the thoughts causes a crisis in rational agency. The suggested treatment can be described as a means to restoring rationality by helping people adopt a more realistic and less pessimistic conception of themselves. Although the strategies offered to people who are struggling with their mental health may differ across various types of interventions, it is often true that such strategies are supposed to remedy a problem that can be characterised as a form of epistemic or practical rationality.

Positive symptoms of schizophrenia include visual and auditory hallucinations and delusions: the person may see things that other people do not see, hear voices other people do not hear, and find reasons for alarm and suspicion in events that do not seem significant to others (e.g., in paranoia). Delusions themselves are defined as irrational beliefs: strong convictions (sometimes with very unusual content) for which the person has no robust evidence and that are not given up in the face of new evidence. In this case, not dissimilar from depression, treatment is judged as effective based on the reduction of the positive symptoms that exemplify a failure of reality testing. Again, the goal can be seen as a restoration of rational behaviour. The desired end is that people can start perceiving and interacting with the world in ways that are shared or at least understandable by people in the same culture.

In a fictional setting, often involving magical creatures or supernatural events, fairytales address situations that are common in our everyday experience, such as power struggles and interpersonal conflicts motivated by greed or jealousy. But even when fairytales reproduce situations we encounter in real life, they often simplify those situations considerably. The witch is evil; the princess is good. There are no grey areas: nothing redeems the witch or contaminates the princess’s virtuous persona. The same applies to the story we have been telling about rationality, agency, and mental health. Rational agency leading to good mental health is what we aspire to.

But there are reasons to believe that agency can be supported by irrational beliefs, that some behaviours associated with mental disorders are the result of coping strategies rather than shortcomings or faults, and that some mental health problems emerge when irrational beliefs are given up (Bortolotti 2020). Such counterintuitive cases are fascinating, leading us to recognise that even behaviours that are described as radical violations of rational norms and as signs of a pathology do not compromise agency but support it at a critical time and contribute to its success. In the rest of the paper, I will offer some examples of such circumstances.

## Sadder but Wiser?

The literature on depressive realism is controversial, due to concerns about the generality and replicability of the results (e.g., Dev et al. 2022). But even accepting its limitations, reflection on the depressive realism effect and on positive illusions tells a different story from the black and white picture where rationality, agency, and mental health are neatly clustered together. What is the effect? Some self-evaluations by people experiencing depressive symptoms seem to be more realistic and better grounded in evidence than self-evaluations by people who do not experience such symptoms (Alloy & Abramson 1979), a phenomenon that has also been called the “sadder but wiser” effect. When we experience low mood and other depressive symptoms, we are less likely to overestimate our capacity to control external events and to erroneously believe that we are above average in our skills, talents, and virtues. This makes us more *epistemically rational* in a specific domain (the domain of accurate self-evaluation) when we experience depressive symptoms. However, the depressive symptoms can have negative repercussions on our mental health.

Why? A pinch of unrealistic optimism may be good for us. It may encourage a distorted version of reality and an inflated conception of ourselves as effective and competent agents, thus compromising our understanding of some situations and leading to unreliable predictions about the future. However, it also fuels the confidence to pursue our goals in the face of adversities and to react positively to setbacks (Taylor 1989). Instead of giving up at the first obstacle or losing faith in ourselves when we receive negative feedback, we find the motivation to persevere with our goals (Bortolotti 2018). That is because, if we believe that we are better than average in most domains and we consider our goals worth pursuing and attainable, then our motivation is less likely to waver.

Various forms of unrealistic optimism have been found to correlate with mastery, self-efficacy, and altruism. And better physical and mental health too. The positive illusions that are so common in the non-clinical population invite us to think of ourselves as:

- more capable to control our external environment than we actually are (illusion of control);
- more skilled and virtuous than average, even when there is no evidence to support this claim to superiority with respect to our peers (illusion of superiority); and
- less likely to experience bad things such as the breakdown of a long-term relationship or serious illness (optimism bias).

When we are not experiencing depressive symptoms, positive illusions have free rein, and we seem to have an excessively rosy picture of what our future holds. We tend to see our life story as characterised by an upward trajectory and a happy ending. When we experience low mood, we do not enjoy the effects of positive illusions to the same extent and thus we are less protected from anxiety and depression. Our mental health is compromised, but not necessarily by pessimism (e.g., excessively negative thoughts) but by realism (e.g., thoughts that do not lead to an overestimation of control, skills, and positive outcomes).

Although the depressive realism effect is limited in scope, as people with depressive symptoms are more realistic at some self-evaluation tasks but not generally, the implications of the depressive realism effect are important. At times, rationality, agency, and mental health do not go hand in hand, and some beliefs that are not well supported by evidence (such as those associated with positive illusions) can contribute to successful agency and protect from mental health issues. Whether specific “sadder but wiser” effects are generalisable or replicable, it does seem plausible that in some circumstances the tendency to be optimistic about ourselves can support agency and mental health even if it leads to irrational beliefs.

Appreciating the dynamic relationship between rationality and agency can help us better understand serious problems that afflict us as individuals and as society and is instrumental to designing more effective solutions. Should we conceive of talking therapies, then, as restoring rationality or as introducing some innocuous, maybe even beneficial, forms of irrationality?

## Delusions as Emergency Responses

It is not hard to accept that some positive thinking can be a boost to agency: when we believe in ourselves, we are better motivated to pursue our goals in the face of obstacles because we think we have a concrete chance at achieving such goals. It is much harder to accept that delusional beliefs may have a beneficial role in supporting agency. Partly, this is because delusions are often perceived as the paradigmatic instance of madness, intended as a form of radical and *disruptive* irrationality. They are often the standard example of a distortion of reality that compromises both our psychological functioning and our understanding of reality. Whereas positive illusions are considered as *barely* irrational, just a positive spin on reality, delusions are conceived as radical departures from reality.

Indeed, when pressed to explain how excessively optimistic beliefs can enhance mental health, Shelley Taylor replies that positive illusions *are not delusions*: they distort reality to a much lesser degree. Taylor’s reply is unsurprising but unsatisfactory: some optimistically unrealistic beliefs are very implausible and difficult to distinguish from prototypical cases of delusions, at least on epistemic grounds (that is, based on whether they reflect how things are and whether they are supported by the evidence available to us). But Taylor’s reply is an indication of the perceived gulf between the innocuous, everyday irrationality of optimistic biases and the dangerous, radical irrationality of delusions. Surely, we can concede that the former have benefits, but to redeem the latter is a different story.

The case of delusional beliefs differs from the case of unrealistically optimistic beliefs: the standard effects of endorsing such beliefs diverge, where the endorsement of delusions is more likely to cause disruption and interfere with functioning. But it is not clear that the increased disruption is due to the delusion departing from reality to a greater extent than the positive illusion. It may be due to the fact that, differently from positive illusions, delusions are more infrequent and heavily stigmatised, thereby leading to the association with negative stereotypes and social exclusion.

My view is that both types of irrational beliefs, positive illusions and delusions, can support agency. In the case of unrealistically optimistic beliefs, the advantage comes from retaining the motivation for pursuing the desired goals in the context of the inevitable setbacks that are part and parcel of imperfectly happy human lives. In the case of the delusional beliefs that we consider symptoms of serious mental illness, instead, the advantage comes from relieving overwhelming anxiety and negative emotions that dramatically impact agency—sometimes paralyse it. In other words, the delusion kicks in at a time of crisis, and should be understood as an emergency response (McKay & Dennett 2009).

Let’s consider the case of anosognosia. In anosognosia (literally, “denial of illness”), the delusion follows a trauma or the onset of an illness or a disability that affects our sense of ourselves as self-sufficient agents. When we experience anosognosia, we refuse to acknowledge either the new impairment or some of its implications for our functioning. For instance, a woman who can no longer move her arm does not admit that her arm is paralysed and claims instead that she can clap her hands, a claim that clashes with the perception of her own movements; a man whose leg has been amputated says that the reason why he finds it hard to climb stairs is that he has arthritis, showing that he recognises his inability to perform certain tasks but is not ready to acknowledge the extent of the disability caused by the amputation (Ramachandran 1996).

V.S. Ramachandran proposes that what causes delusions to emerge is an exaggerated version of our normal defence mechanisms that have an adaptive function: by denying the recent changes, we maintain a coherent picture of ourselves as we were before the impairment (our “premorbid self”). The delusion can be seen as a way to preserve continuity by failing to update personal information and deal better with the loss of previous social roles due to illness. Anosognosia can, of course, be harmful: if we do not acknowledge that we have an illness or a disability, we may refuse to seek treatment or fail to engage in rehabilitation. But some positive effects of anosognosia on wellbeing are reported too: the presence of anosognosic delusions is associated with fewer negative emotions and reduced anxiety (Aimola Davies et al. 2009).

One interesting illustration of how rationality and mental health can come apart is offered by the *insight paradox*. The paradox emerges from the observation that in schizophrenia, contrary to expectations, when we start realising the delusional nature of our beliefs, we risk experiencing very severe depression and even suicidal thoughts (Bortolotti & Belvederi Murri 2025). With better insight, we become aware that we did not have a good grip on reality, and may be overwhelmed by feelings of shame and sadness (Belvederi Murri & Amore 2018). Realising that we have been suffering from a mental illness can also make us feel hopeless, and our self-esteem plummets. That is why insight has been characterised as “a double-edged sword”: if better insight means that we have more accurate beliefs and yet lose self-esteem, low insight can be conceived as a way of coping.

## Conclusions and Implications

Based on this brief overview of some significant cases of “helpful” irrational beliefs, we can start sketching a complex picture of human cognition and agency, where rationality gains can have bleak implications for mental health, and departures from consensual reality or accurate representations of reality can make us more resilient and successful, at least in some contexts. This is not the traditional fairytale: the princess may be ugly, and the witch may be brave, after all. But this unorthodox story, this twisted fairytale, can help us understand human agency better.

How? Reflecting on the delicate balance between following the evidence and pursuing happiness can engender a better knowledge of ourselves and a greater appreciation of the strategies we use to explain the world around us. It is important to realise that the pursuit of knowledge itself wouldn’t be possible if we could not count on a sustained motivation to chase our goals, which include our goal to attain knowledge. Our strategies have multiple objectives, often pulling in different directions: they need to help us build a map of reality that is as accurate as possible, but also to preserve a sense of competence and efficacy that enables us to use that map with confidence.

The realisation that our sense of ourselves as competent agents is essential to agency and mental health, even when it is partially illusory, can support us in one of the most challenging tasks we encounter, the respectful and productive interaction with agents who have radically different beliefs and values. For instance, how should we approach exchanges with people who endorse non-mainstream explanations for the significant events that impact our lives? The traditional picture where rationality is a precondition for agency could tempt us to dismiss their views as unfounded (or worse), and more generally exclude them from shared epistemic projects. We may not be inclined to include them in collective decision making and problem solving, based on concerns about their capacity to exercise agency.

But lack of engagement may turn out to be a mistake, as the endorsement of irrational beliefs, or at least the endorsement of beliefs that we do not share, may be an expression of agency as opposed to a symptom of compromised agency. Of course, we may have reasons not to engage with a person’s views independently of an assessment of their capacity for agency. For instance, we may believe that the person does not sincerely subscribe to those views, and we conclude that our engagement would not be productive for either party. Alternatively, we may be concerned that engaging with those views in a certain context would cause significant harm to ourselves or others, as in the case of racial attacks or hate speech. In such cases, even if a person’s views are a genuine expression of their agency, engagement may not be advisable.

What I am suggesting here is that the presence of different beliefs and values by itself does not justify lack of engagement. Hopefully, a better awareness of the multiple factors contributing to our mental life, including the imperfect theories, solutions, and decisions we arrive at, helps us see that what appears as a person’s failure of rationality or departure from reality does not necessarily compromise their capacity to pursue their goals. Neither does it disqualify them from productive exchanges, where we learn something new even if we are unlikely to change our minds.
